# Supplementary material for: A unifying model to explain frequent SARS-CoV-2 rebound after nirmatrelvir treatment and limited prophylactic efficacy
Source: Nat Commun. 2024 Jun 28;15:5478. doi: 10.1038/s41467-024-49458-9 (PMC11213957; doi:10.1038/s41467-024-49458-9)
Supplement: Supplementary file 1 — Supplementary Information [file 41467_2024_49458_MOESM1_ESM.pdf]

Supplementary Materials for

A unifying model to explain frequent SARS-CoV-2 rebound after nirmatrelvir treatment and limited prophylactic efficacy

Shadisadat Esmaeili *et al*.

*Corresponding author. Email: sesmaeil@fredhutch.org

This file includes:

Figs. S1 to S16
Tables S1 to S5

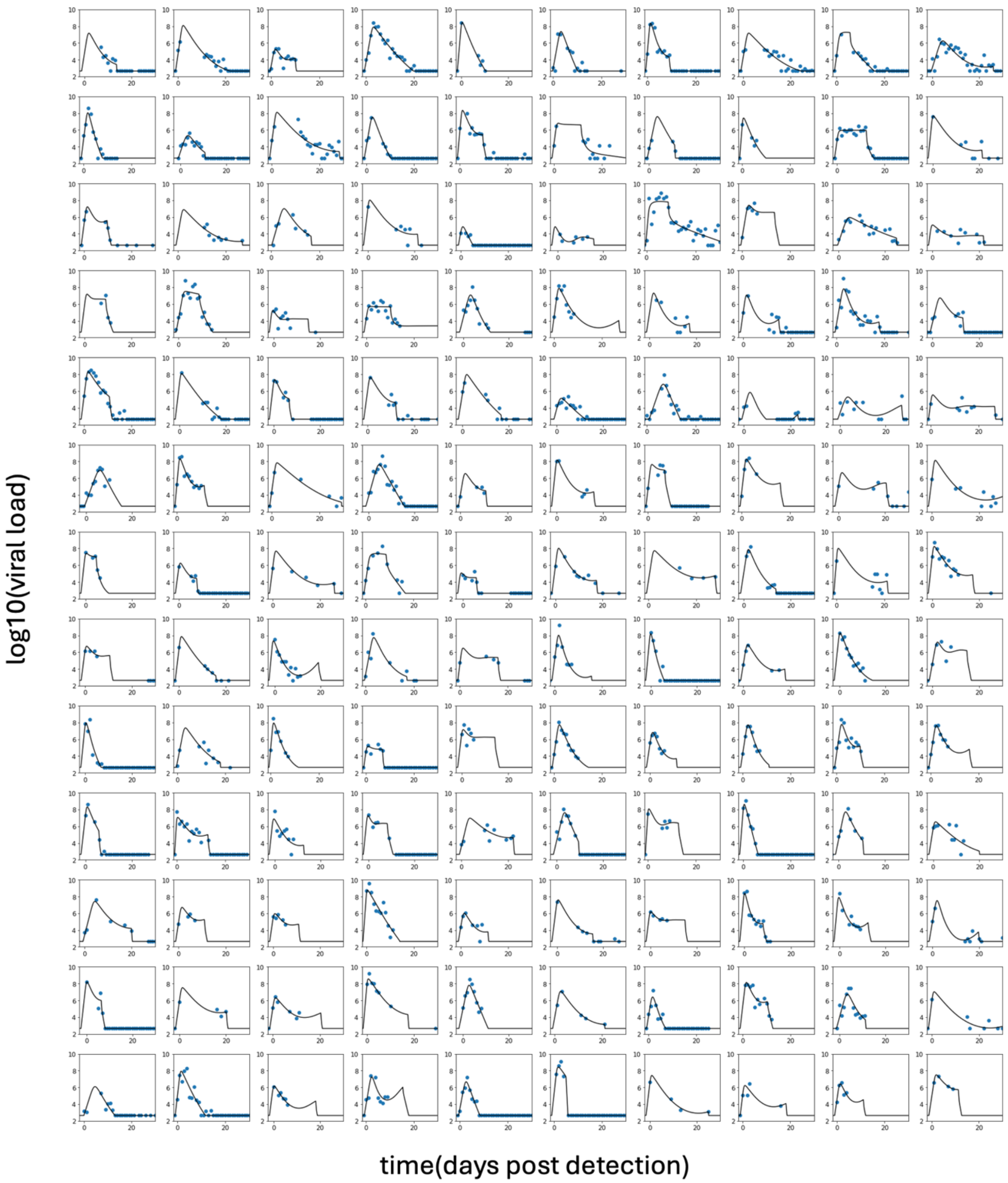

Fig S1. Viral dynamics model recapitulation of untreated SARS-CoV-2 kinetics. Viral dynamics model fit to diverse individual data. Included panels are 130 sample individuals from the NBA cohort with positive symptom status.

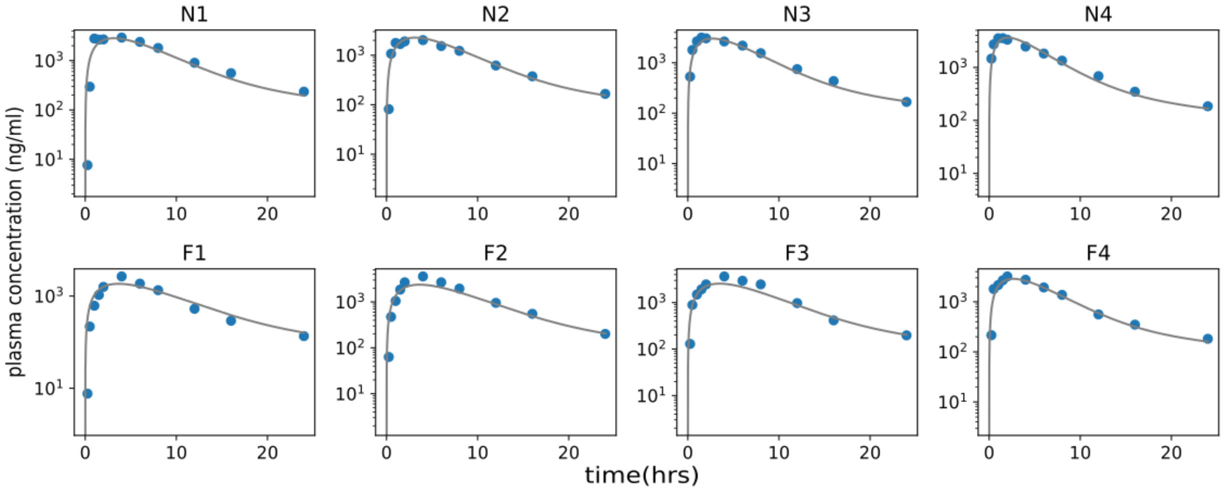

Fig S2. Pharmacokinetic (PK) model recapitulation of plasma concentration of nirmatrelvir. PK model fits individual nirmatrelvir/ritonavir plasma concentration data of 8 individuals (4 fed (F), 4 fasting (N)) with the 250 mg/100 mg dose of nirmatrelvir/ritonavir. Fed and fasting refer to individuals taking the drug under fed and fasting conditions, as explained in the original study by Singh et al (2022).

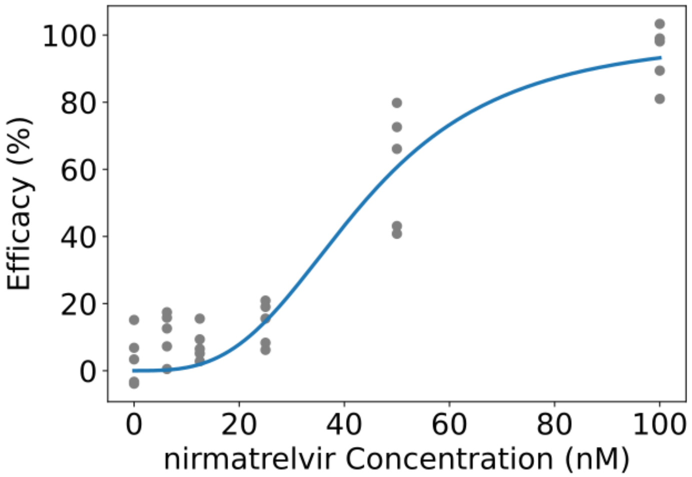

Fig S3. Pharmacodynamic (PD) model fitting to mean antiviral efficacy as a function of drug concentration in vitro. PD model fitting to mean efficacy of nirmatrelvir+efflux inhibitor was obtained from 5 replicates pooled from 2 in vitro infection experiments in Calu3 cells using the least square method.

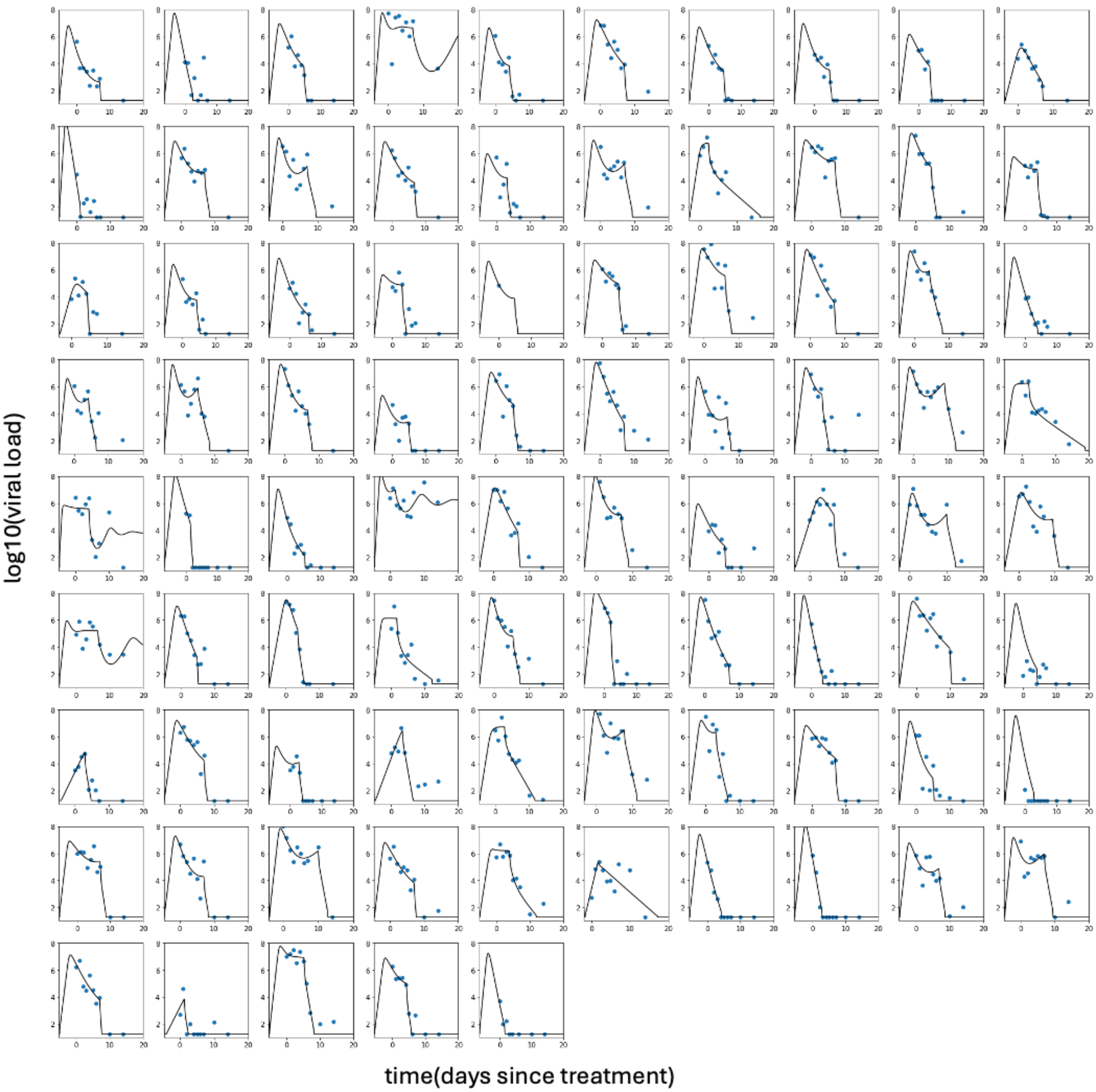

Fig S4. Mathematical model recapitulation of the control arm of the PLATCOV trial.
Combined model fit to individual data.

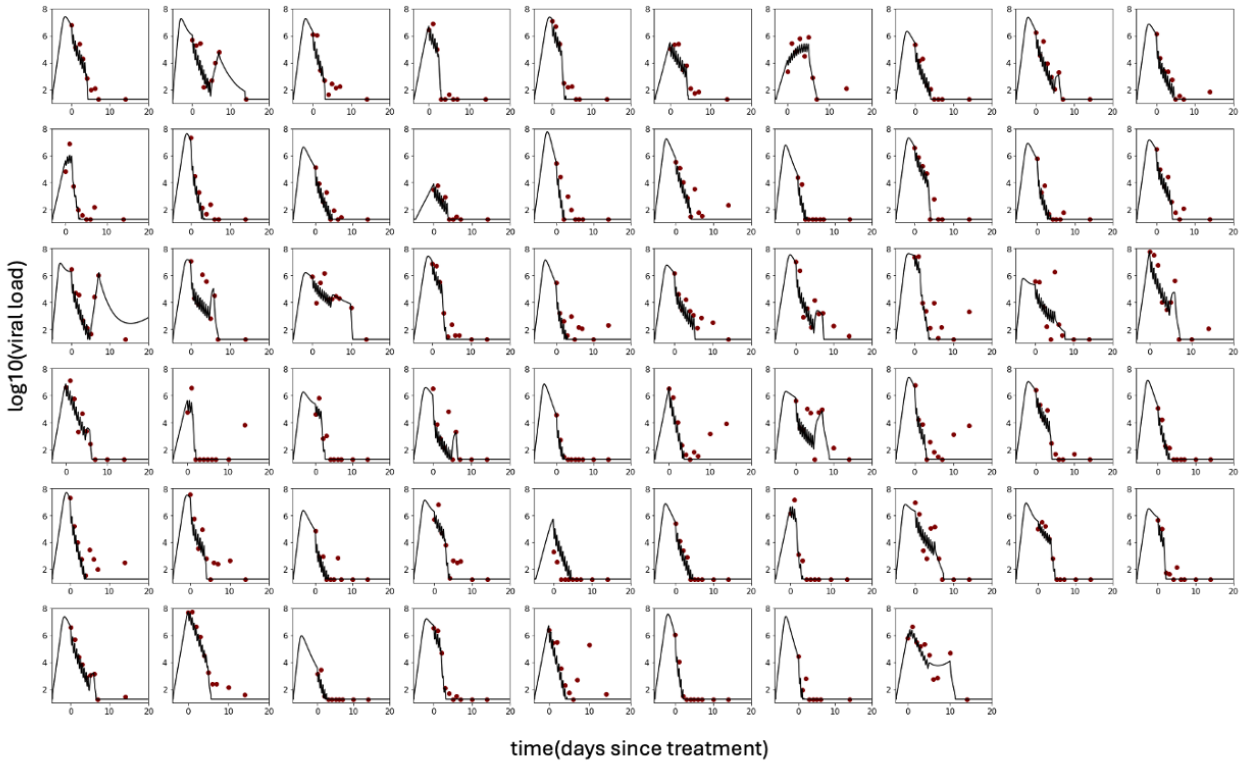

Fig S5. Mathematical model recapitulation of the treatment arm of the PLATCOV trial. Combined model fit to individual viral load data. PK and PD parameters were fixed for all individuals.

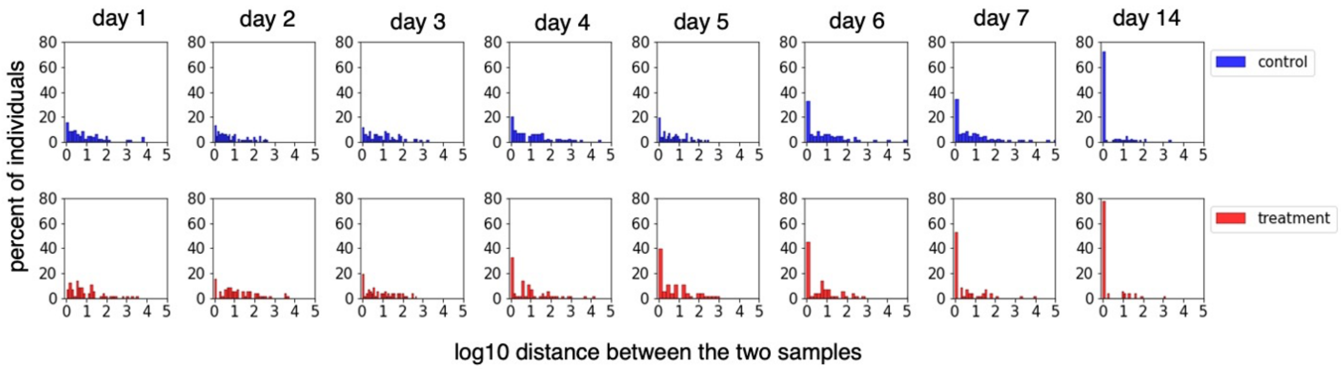

Fig S6. Differences in SARS-CoV-2 viral loads from contemporaneous oral samples collected from each individual in the PLATCOV trial *(25)*. The distribution of distance between the two samples collected from each participant's left and right tonsil in the control arm (top row) and the treatment arm (bottom row). In certain instances, there is a substantial difference in viral loads between the two samples.

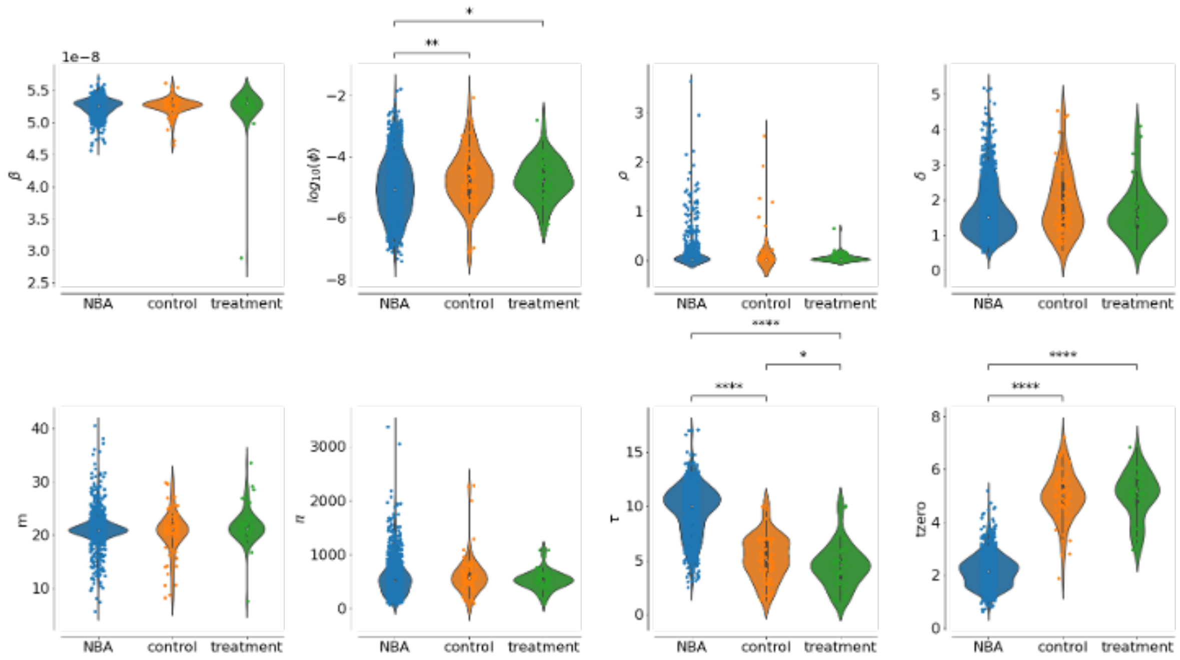

Fig S7. Population parameter distributions for symptomatic Omicron-infected individuals of NBA cohort and the two arms of the PLATCOV trial. In the PLATCOV trial τ and t_{zero} are estimated relative to the time of the baseline measurement, while in the NBA cohort, they are estimated relative to the first positive test. p-values were obtained by performing two-sided Mann-Whitney U-test ($*: 0.01 < p \leq 0.05$,$**: 0.001 < p \leq 0.01$,$***: 0.0001 < p \leq 0.001$,$****: 0.00001 < p \leq 0.0001$).

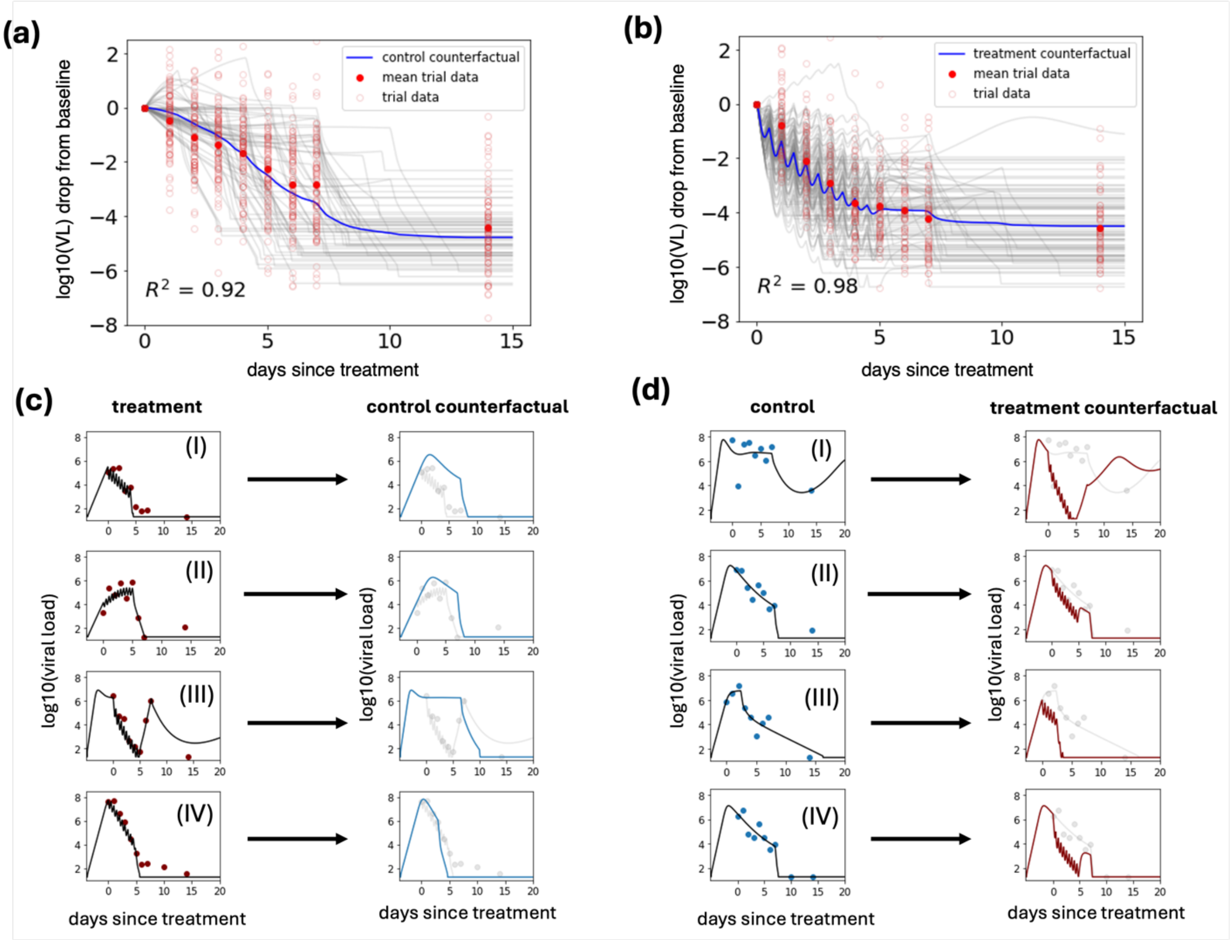

Fig S8. Counterfactual simulations of the control and treatment arms of the PLATCOV trial recapitulate trial outcomes. (a-b) individual simulated control counterfactual of the treatment arm (n=58) **(a)** and the treatment counterfactual of the control arm (n=85) **(b)** (in grey) and the mean viral load drop from the baseline (in blue) and the trial data. **(c-d)** sample model fits to the treatment arm and its control counterfactual simulation **(c)** and control arm and its treatment counterfactual simulation**(d)**.

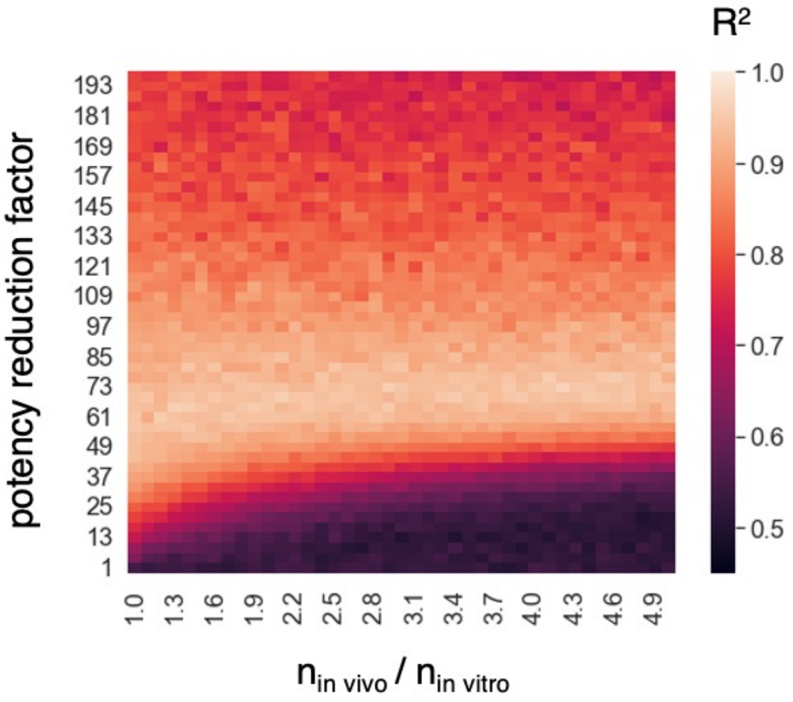

Fig S9. The estimated potency reduction factor is independent of the in vivo Hill coefficient. The R^2 of the fit to the treatment arm of EPIC-HR for different prf values and Hill coefficients. The best fit with the highest R^2 is obtained for prf ~ 60 and is independent of the Hill coefficient.

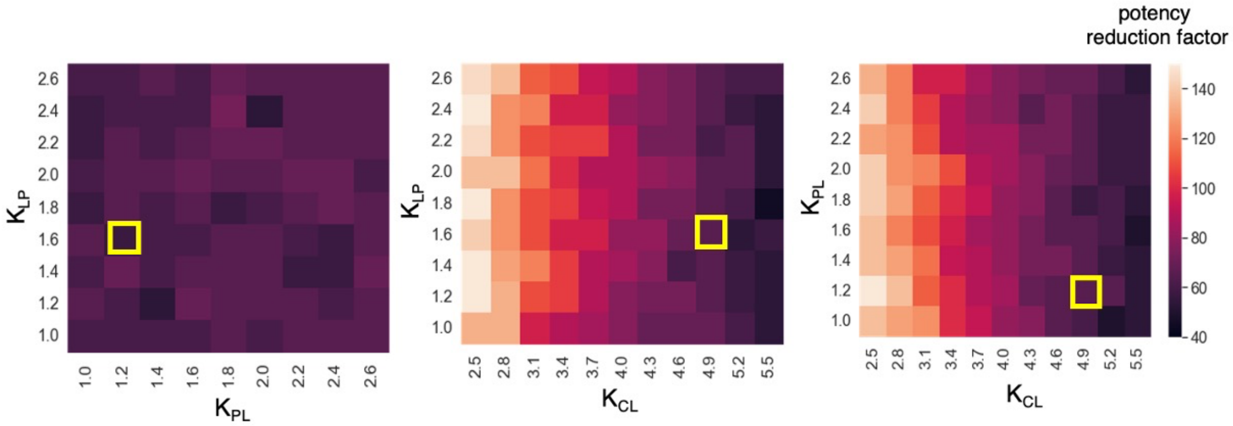

Fig S10. Only the drug's clearance rate affects the estimated potency reduction factor. Potency reduction factor sensitivity to PK parameters fit to data from EPIC-HR. The yellow square in each panel marks the estimated values of the PK parameters by fitting the PK model to the trial data with single dose of 250mg/100mg nirmatrelvir/ritonavir.

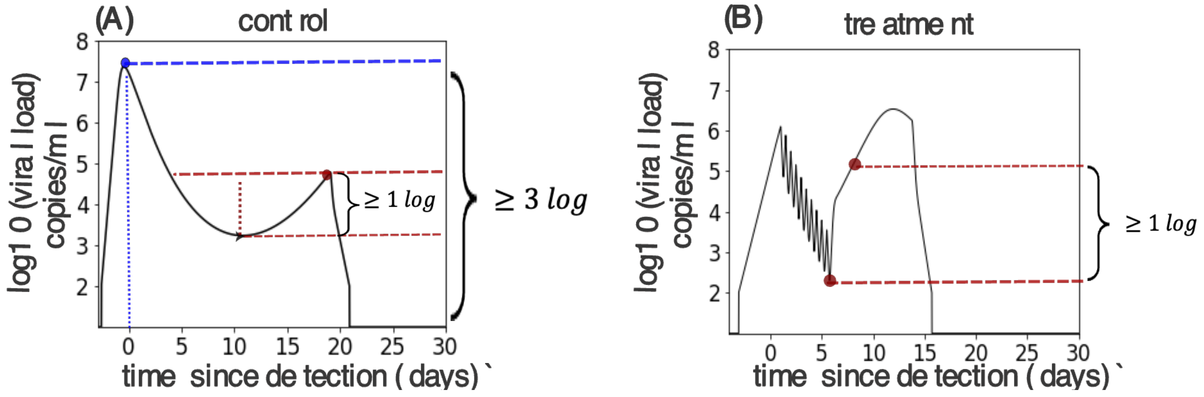

Fig S11. Rebound definition in control and treatment arms. (A) Rebound was flagged in the control arm if there were two peaks with a height greater than 3 logs and the second peak higher than the post-peak trough by at least 1 log. (B) Rebound in treatment arm was defined as increased viral load by at least 1 log any time after the end of treatment.

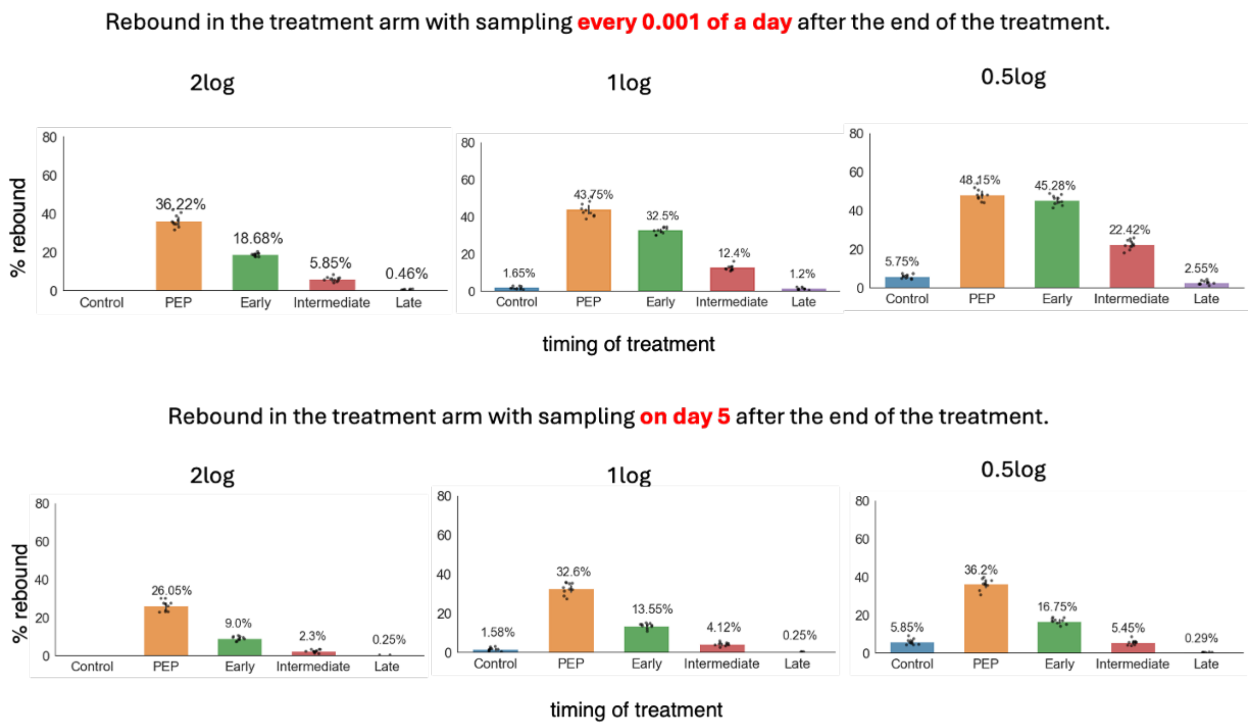

Fig S12. Sensitivity of viral rebound detection to rebound definition and sampling frequency. Top row: rebound probability in the treatment arm measured by sampling every 0.001 of a day after treatment and defining rebound as cases when viral load exceeds the viral load at the end of the treatment by 2 log, 1 log, or 0.5 log (left to right). Bottom row: rebound probability measured by sampling on day 5 after treatment ends with the same thresholds as the top row from left to right. In all panels, the error bars show the 95% confidence interval obtained by running the simulation on 400 people 10 times. The data points on each bar shows the probability obtained from each simulation. The height of each bar equals the mean of the 10 data points.

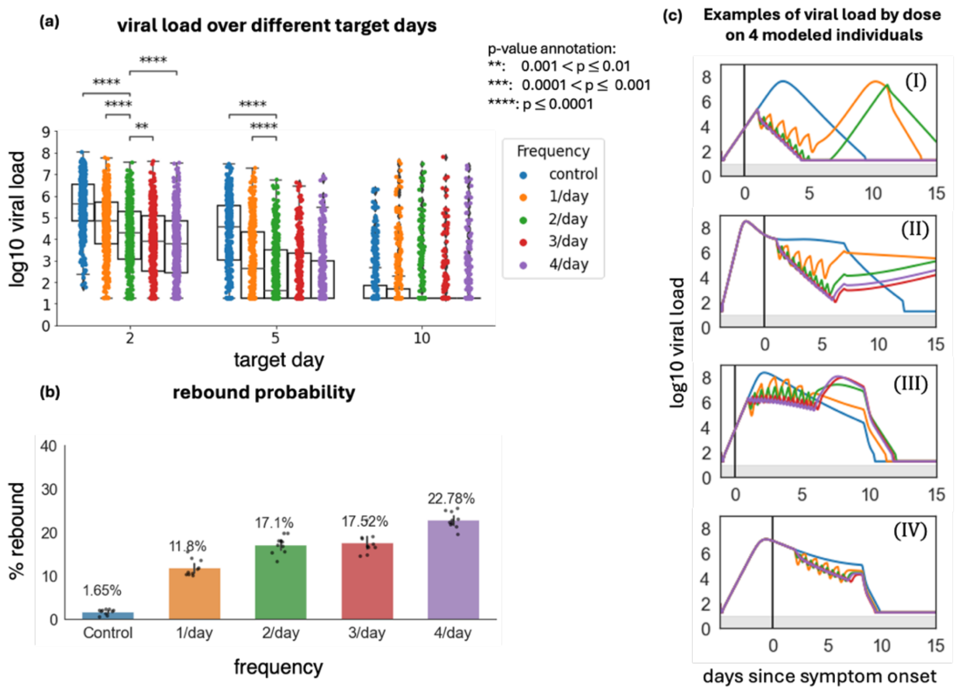

Fig. S13. Increasing nirmatrelvir dosing frequency lowers short term viral load but increases probability of viral rebound. In all simulations, 300 mg treatment starts within the first 3 days post-symptoms. **(a)** log10 viral load of 400 individuals (n=400) at days 2, 5, and 10 after the treatment start day with different dosing frequencies. p-values are obtained by performing two-sided Mann-Whitney U-test between 2/day group and others and only values <0.01 are shown. Viral loads are only reduced by higher dosing frequency at days 2 and 5, but not day 10. **(b)** The probability of rebound for different doses. The error bars on each column are 95% confidence interval obtained by running the simulation on 400 people 10 times. The data points on each bar shows the probability obtained from each simulation. The height of each bar equals the mean of the 10 data points. **(c)** Samples of viral load trajectories assuming different dosing frequency on 4 modeled individuals with equivalent timing of therapy and untreated viral kinetics.

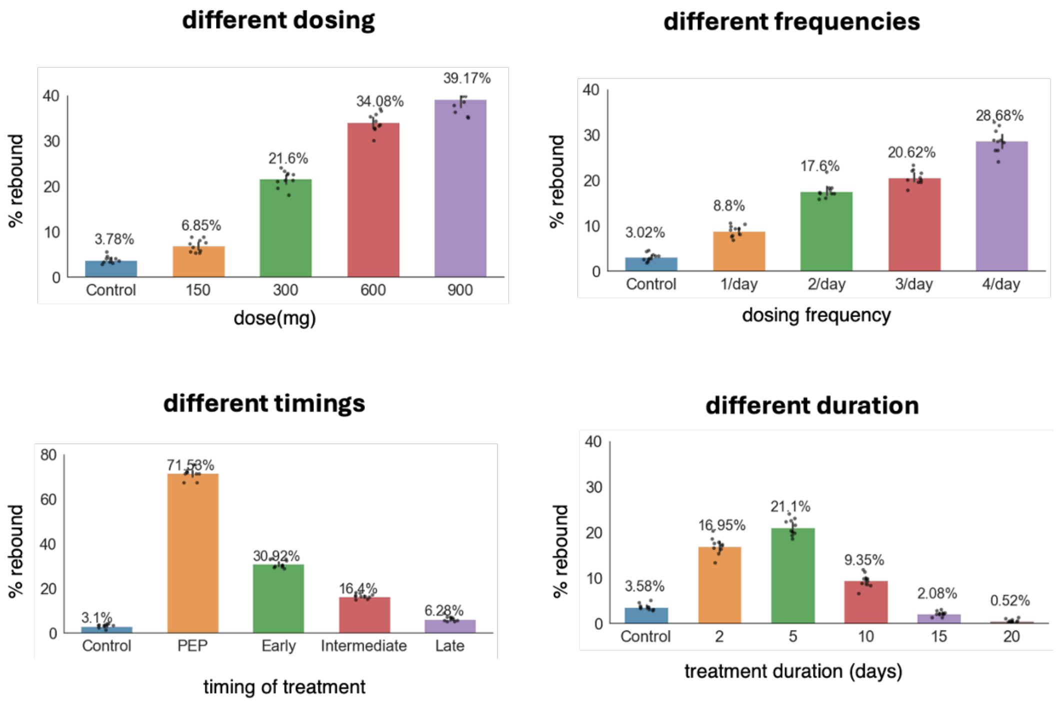

Fig. S14. Higher probabilities of viral rebound among unvaccinated individuals mostly infected with pre_Omicron VOC. In the control arm and all treatment scenarios the model predicts higher incidents of rebound in the unvaccinated pre_Omicron individuals. These results can be compared to **Figs 4-7 and Fig S13** and show consistently higher rebound assuming equivalent conditions. In all panels, the error bars show the 95% confidence interval obtained by running the simulation on 400 people 10 times. The data points on each bar shows the probability obtained from each simulation. The height of each bar equals the mean of the 10 data points.

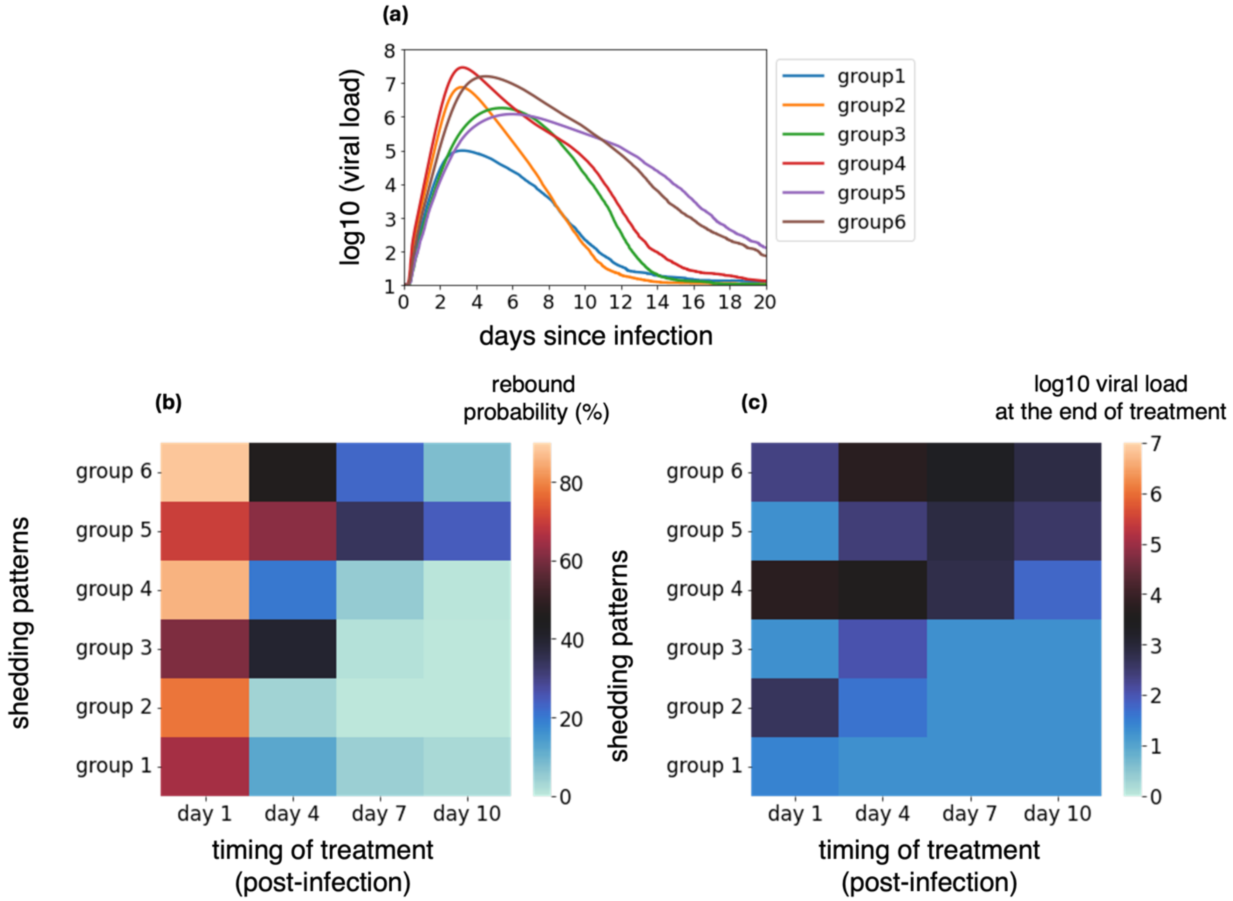

Fig S15. Higher rebound probability in individuals with higher peak viral load and faster viral expansion after early treatment. (a) Modeled mean viral load trajectories of six different groups off-treatment demonstrating differing peak viral load, time of viral peak, clearance rate, and time to clearance. Shedding pattern groups were from the NBA cohort identified in Owens et al. using k-means clustering. Higher group number indicates a higher viral area under the curve assuming untreated infection. **(b)** The probability of rebound and **(c)** the viral load at the end of treatment with different timing of treatment initiation. (Number of individuals in each group: n_1=115, n_2=168, n_3=155, n_4 = 154, n_5=74, n_6=102).

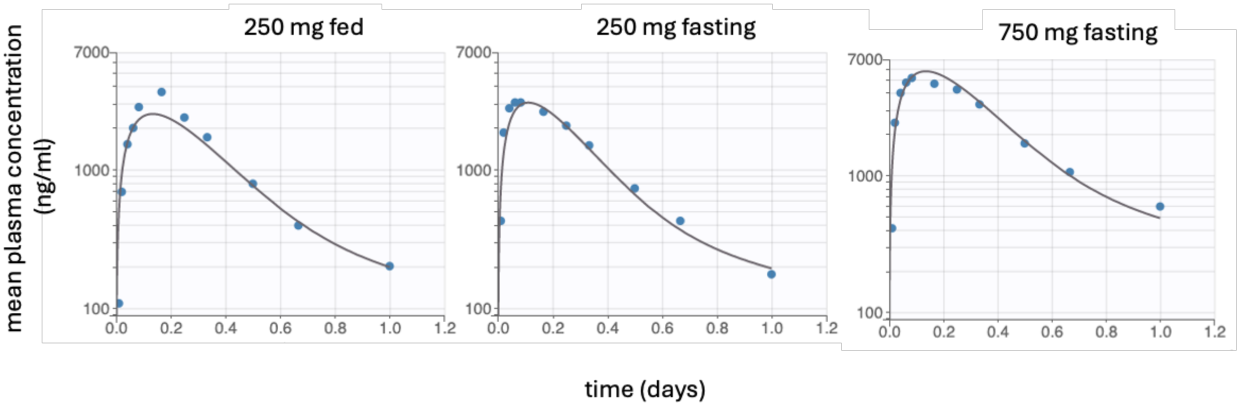

Fig S16. Pharmacokinetic (PK) model recapitulation of mean plasma concentration of nirmatrelvir with different doses. PK model fits mean nirmatrelvir/ritonavir plasma concentration data for three groups with 250 mg/100 mg dose of nirmatrelvir/ritonavir (fed), 250 mg/ 100 mg (fasting), and 750 mg /100 mg (fasting). Fed and fasting refer to individuals taking the drug under fed and fasting conditions, as explained in the original study by Singh et al (2022).

Parameter (unit)	Symbol	Population Mean	Standard error	Standard deviation of random effects	Distribution	Source
viral infectivity ($\log_{10}$ (RNAcopies/mL)$^{-1}$ day^{-1})	$\log_{10}\beta$	-7.31	3.17e-3	0.047	normal	estimated
viral production rate ($\log_{10}$ day^{-1})	$\log_{10}\pi$	2.74	9.7e-3	0.316	normal	estimated
rate at which refractory cells revert to susceptible state ($\log_{10}$ day^{-1})	$\log_{10}\rho$	-1.62	3.33e-2	0.972	normal	estimated
rate constant for conversion of target cells to a refractory state ($\log_{10}$ cell^{-1}day^{-1})	$\log_{10}\phi$	-5.29	6.52e-2	1.2	normal	estimated
Deviation from $\log_{10}\phi$ for delta/other	$\beta_{\phi_{omicron}}$	0.324	7.38e-2	--	--	--
infected cell clearance rate (day^{-1} cells^{-1})	δ	1.38	2.64e-2	0.565	lognormal	estimated
onset of acquired immunity relative to detection (days)	τ	15.9	0.453	0.461	lognormal	estimated
Deviation from τ for delta/other	$\beta_{\tau\ omicron}$	-0.364	3.53e-2	--	--	--
Deviation from τ for unvaccinated/no record	$\beta_{\tau\_>1vax}$	-0.141	3.16e-2	--	--	--
Increase in clearance rate of infected cells due to acquired immunity (day^{-1})	m	16.4	0.542	0.502	lognormal	estimated
delay between infection in nasal tissue and detection (days)	t_0	2.08	2.8e-2	0.463	logit[0,20]	estimated
initial viral inoculum (RNAcopies/mL)	V_0	97	--	--	--	fixed
viral clearance rate (day^{-1})	γ	15	--	--	--	Goyal et al.
mean eclipse phase duration (days^{-1})	$1/k$	1/4	--	--	--	Ke et al.
Initial number of susceptible cells	S_0	1×10^7	--	--	--	Ortiz et al.
Initial number of refractory cells	R_0	0	--	--	--	--
Initial number of productively infected cells	$I_{P,0}$	0	--	--	--	--
Initial number of infected cells in eclipse phase	$I_{E,0}$	0	--	--	--	--

Table S1. Population parameters of viral dynamics model. Using the viral dynamic model we estimated model parameters for 1510 infections in the NBA cohort. For the mixed-effect model, a constant error model was used, and the magnitude of measurement error was fixed at a = 0.25 log10 copies viral RNA/ml. Vaccine status (0 vs >1 dose) was set as a covariate on τ, and the variance (other/delta vs omicron) was set as a covariate for τ and ϕ. Also, linear dependencies between (π, ϕ, δ) and (τ, ρ) were set up in Monolix. The population parameters are recorded here and estimated individual parameters are available at https://github.com/sEsmaeili/Covid_Rebound.

Parameter (unit)	Symbol	Population Mean	Standard deviation	Distribution
Absorption rate (day^{-1})	κ_a	9.98	0.37	lognormal
Rate of transfer from plasma to lung (day^{-1})	κ_{PL}	1.58	0.1	lognormal
Rate of transfer from lung to plasma (day^{-1})	κ_{LP}	1.22	0.1	lognormal
Clearance rate (day^{-1})	κ_{CL}	4.96	0.03	lognormal
Plasma Volume (ml)	Vol	41743	0.12	lognormal
Error parameter	b	0.34		

Table S2. Population parameters of pharmacokinetic model. Using the two-compartmental PK model with oral administration of the drug, and proportional error model (estimated error parameter b=0.34), we estimated model parameters for 8 healthy individuals in the phase I randomized clinical trial by Singh et al. (2022) .The population parameters are recorded here and estimated individual parameters are available at https://github.com/sEsmaeili/Covid_Rebound.

	κ_a	κ_{LP}	κ_{CL}	κ_{PL}	Vol
250(fed)	8.81	1.28	4.77	1.72	42571.61
250(fasting)	12.32	1.28	4.78	1.76	40379.71
750(fasting)	8.49	1.29	4.78	1.92	54274.78

Table S3. Population parameters of pharmacokinetic model for different doses. Similar PK parameters were estimated for different doses by fitting the two-compartmental PK model with oral administration of the drug to population-level plasma concentrations at different doses. The variability in κ_a is also reflected in the individual parameter values estimated for the 250mg, fed, and fasting groups, and therefore, it is not obvious whether it is dose-dependent. Fed and fasting refer to individuals who took the drug fed or fasting conditions as explained in the original study by Singh et al (2022).

Parameter (unit)	Symbol	Mean	Standard Error
Maximum efficacy (%)	E_{max}	99.9	0.026
Drug concentration to provide 50% efficacy ($nMol$)	IC_{50}	43.6	3.2
Hill coefficient	n	3.16	0.42

Table S4. Pharmacodynamic parameters. Using the least square method, we estimated the PD parameters by fitting the Hill equation to the in vitro data.

Parameter (unit)	Symbol	Population Mean	Standard error	Std dev. of random effects	Distribution	Source
viral infectivity (log_{10} (RNAcopies/mL)$^{-1}$ day^{-1})	$\log_{10}\beta$	-7.28	4.23e-3	5.66e-2	normal	estimated
viral production rate (log_{10} day^{-1})	$\log_{10}\pi$	2.72	1.12e-2	0.311	normal	estimated
rate at which refractory cells revert to susceptible state (log_{10} day^{-1})	$\log_{10}\rho$	-1.57	3.87e-2	0.932	normal	estimated
rate constant for conversion of target cells to a refractory state (log_{10} cell^{-1}day^{-1})	$\log_{10}\phi$	-5.01	4.05e-2	1.18	normal	estimated
infected cell clearance rate (day^{-1} cells^{-1})	δ	1.49	3.36e-2	0.556	lognormal	estimated
onset of acquired immunity relative to detection (days)	τ	10.8	0.405	0.473	lognormal	estimated
Deviation from τ for unvaccinated/no record	$\beta_{\tau\_>1vax}$	-0.057	--	--	--	--
Deviation from τ for NBA Omicron Individuals	$\beta_{\tau\_PLATCOV}$	-0.831	--	--	--	--
Increase in clearance rate of infected cells due to acquired immunity (day^{-1})	m	20.9	0.953	0.548	lognormal	estimated
time of infection relative to first detection (days)	t_0	2.13	3.46e-2	0.456	logit[0,20]	estimated
Deviation from t_0 for NBA	$\beta_{t_0,PLATCOV}$	1.05	6.32e-2	--	--	--
Potency reduction factor for PLATCOV treatment arm	prf	40.3	5.48	0.826	lognormal	estimated
initial viral inoculum (RNAcopies/mL)	V_0	97		--	--	fixed
viral clearance rate (day^{-1})	γ	15		--	--	Goyal et al.
mean eclipse phase duration (days^{-1})	$1/k$	1/4		--	--	Ke et al.
Initial number of susceptible cells	S_0	1×10^7		--	--	Ortiz et al.
Initial number of refractory cells	R_0	0		--	--	--
Initial number of productively infected cells	$I_{P,0}$	0		--	--	--
Initial number of infected cells in eclipse phase	$I_{E,0}$	0		--	--	--

Table S5. Population parameters of combined viral dynamics + PKPD model fit to PLATCOV + NBA Omicron data. Using the viral dynamic+PKPD model we estimated model parameters for Omicron infections in the NBA cohort and control and treatment arms of PLATCOV trial. For the mixed-effect model, a constant error model was used, and the magnitude of measurement error was fixed at a = 0.25 log10 copies viral RNA/ml. Vaccine status (0 vs >1 dose) was set as a covariate on τ, and the cohort (NBA vs PLATCOV) was set as a covariate for τ and t_0. Also, linear dependencies between (π, ϕ, δ) and (τ, ρ) were set up in Monolix. The population parameters are recorded here and estimated individual parameters are available at https://github.com/sEsmaeili/Covid_Rebound.
