# Supplementary material for: A unifying model to explain frequent SARS-CoV-2 rebound after nirmatrelvir treatment and limited prophylactic efficacy
Source: Nat Commun. 2024 Jun 28;15:5478. doi: 10.1038/s41467-024-49458-9 (PMC11213957; doi:10.1038/s41467-024-49458-9)
Supplement: Supplementary file 3 — Reporting Summary [file 41467_2024_49458_MOESM3_ESM.pdf]

Corresponding author(s): Shadisadat Esmaeili-Wellman

Last updated by author(s): May 17, 2024

Reporting Summary

Nature Portfolio wishes to improve the reproducibility of the work that we publish. This form provides structure for consistency and transparency in reporting. For further information on Nature Portfolio policies, see our Editorial Policies and the Editorial Policy Checklist.

Statistics

For all statistical analyses, confirm that the following items are present in the figure legend, table legend, main text, or Methods section.

n/a	Confirmed	
☐	☒	The exact sample size (*n*) for each experimental group/condition, given as a discrete number and unit of measurement
☐	☒	A statement on whether measurements were taken from distinct samples or whether the same sample was measured repeatedly
☐	☒	The statistical test(s) used AND whether they are one- or two-sided<br>*Only common tests should be described solely by name; describe more complex techniques in the Methods section.*
☐	☒	A description of all covariates tested
☐	☒	A description of any assumptions or corrections, such as tests of normality and adjustment for multiple comparisons
☐	☒	A full description of the statistical parameters including central tendency (e.g. means) or other basic estimates (e.g. regression coefficient) AND variation (e.g. standard deviation) or associated estimates of uncertainty (e.g. confidence intervals)
☐	☒	For null hypothesis testing, the test statistic (e.g. *F*, *t*, *r*) with confidence intervals, effect sizes, degrees of freedom and *P* value noted<br>*Give P values as exact values whenever suitable.*
☒	☐	For Bayesian analysis, information on the choice of priors and Markov chain Monte Carlo settings
☒	☐	For hierarchical and complex designs, identification of the appropriate level for tests and full reporting of outcomes
☒	☐	Estimates of effect sizes (e.g. Cohen's *d*, Pearson's *r*), indicating how they were calculated

Our web collection on statistics for biologists contains articles on many of the points above.

Software and code

Policy information about availability of computer code

Data collection	Phramacokinetics data used in this paper were digitized using web plot digitizer (https://automeris.io/WebPlotDigitizer.html).
Data analysis	Mode fitting was performed using Monolix version 2023R1<br>Data analysis and simulations were performed in Python 3.9.12<br>All costume codes written in python are shared in Github: https://github.com/sEsmaeili/Covid_Rebound

For manuscripts utilizing custom algorithms or software that are central to the research but not yet described in published literature, software must be made available to editors and reviewers. We strongly encourage code deposition in a community repository (e.g. GitHub). See the Nature Portfolio guidelines for submitting code & software for further information.

Data

Policy information about availability of data

All manuscripts must include a data availability statement. This statement should provide the following information, where applicable:

- Accession codes, unique identifiers, or web links for publicly available datasets
- A description of any restrictions on data availability
- For clinical datasets or third party data, please ensure that the statement adheres to our policy

The data analyzed in this work was previously published by Hay et al. and Schilling et al. and is available on github at https://github.com/gradlab/SC2-kinetics-immune-history and https://github.com/jwatowatson/PLATCOV-Molnupiravir/tree/V1.0

The pharmacodynamics data provided in GitHub: https://github.com/sEsmaeili/Covid_Rebound

Research involving human participants, their data, or biological material

Policy information about studies with human participants or human data. See also policy information about sex, gender (identity/presentation), and sexual orientation and race, ethnicity and racism.

Reporting on sex and gender	In this study we did not collect any new human data.
Reporting on race, ethnicity, or other socially relevant groupings	race and ethnicity of the participants were collected in the original studies but were not used as a covariate in this paper's analysis.
Population characteristics	health status (high-risk vs low-risk individuals as defined by having at least one risk factor for SARS-CoV-2) and vaccination status were used as a covariate in this study
Recruitment	No human participant recruited in this study.
Ethics oversight	No ethincs oversight required due to no new human data collection and human participants recruitment.

Note that full information on the approval of the study protocol must also be provided in the manuscript.

Field-specific reporting

Please select the one below that is the best fit for your research. If you are not sure, read the appropriate sections before making your selection.

☒ Life sciences ☐ Behavioural & social sciences ☐ Ecological, evolutionary & environmental sciences

For a reference copy of the document with all sections, see nature.com/documents/nr-reporting-summary-flat.pdf

Life sciences study design

All studies must disclose on these points even when the disclosure is negative.

Sample size	No sample size calculation was performed. A sample size of n=400 (out of 822 vaccinated individuals with Omicron infection) was used to avoid overlap between virtual cohorts used in each arm of the simulations and between different simulations.
Data exclusions	no data was excluded in this study.
Replication	prf estimation was validated by fitting the model to the results of the two different trials and using two methods for one of the trials (PLATCOV). The results were consistent between the two methods and the difference between the two prf esimatates for the two trials can be attributed to different population characteristics and sampling methods.<br><br>The simulations were repeated 10 times to assure results reproducibility in the presence of different sources of stochasticity (individual viral dynamics parameters, individual PK parameters, individual incubation periods, and individual prf values). the results were qualitatively consistent and the error bars representing the 95% CI of the 10 runs are shown in the figures where applicable.
Randomization	randomization in the context of this study is applied to the randomization of model parameters of each individual. Individual viral load parameters were randomly selected from the NBA cohort. All other parameters were randomly drawn from their estimated population distributions inferred from data.
Blinding	In this study we worked with previously published blinded data.

Reporting for specific materials, systems and methods

We require information from authors about some types of materials, experimental systems and methods used in many studies. Here, indicate whether each material, system or method listed is relevant to your study. If you are not sure if a list item applies to your research, read the appropriate section before selecting a response.

Materials & experimental systems

n/a	Involved in the study
☒	☐ Antibodies
☒	☐ Eukaryotic cell lines
☒	☐ Palaeontology and archaeology
☒	☐ Animals and other organisms
☒	☐ Clinical data
☒	☐ Dual use research of concern
☒	☐ Plants

Methods

n/a	Involved in the study
☒	☐ ChIP-seq
☒	☐ Flow cytometry
☒	☐ MRI-based neuroimaging

Plants

Seed stocks	no plant data used in this study.
Novel plant genotypes	no plant data used in this study
Authentication	no plant data used in this study.
